# Supplementary material for: eDNA Increases the Detectability of Ranavirus Infection in an Alpine Amphibian Population
Source: Viruses. 2019 Jun 6;11(6):526. doi: 10.3390/v11060526 (PMC6631829; doi:10.3390/v11060526)
Supplement: Supplementary file 1 [file viruses-11-00526-s001.pdf]

Supplementary material Table S1. Detection of Ranavirus in common frog (adult and tadpole) at the Balaour pond (Southeaster Alps) in 2016

.

	Tadpole alive		Tadpole dead		Adult alive		Adult dead	
	GE	prevalence	GE	prevalence	GE	prevalence	GE	prevalence
06/10-11	3.97	1/5			0	0/5	$2.83*10^{1}$	1/1
	0				0			
	0				0			
	0				0			
	0				0			
06/23-24	$1.67*10^{1}$	4/5			1.13	3/5		
	$1.33*10^{2}$				$5.98*10^{-1}$			
	8.64				3.89			
	$3.47*10^{1}$				0			
	0				0			
07/08	2.3	5/5			$1.21*10^{3}$	4/5		
	$8.4 *10^{-1}$				$3.91*10^{1}$			
	$1.23*10^{1}$				$4.37*10^{2}$			
	1.76				$2.24*10^{1}$			
	$1.60*10^{1}$							
07/27	$8.4 *10^{-1}$	4/5	$8.9*10^{7}$	10/10				
	$1.23*10^{1}$		$4.48*10^{6}$					
	1.76		$1.58*10^{7}$					
	$1.60*10^{1}$		$5.38*10^{6}$					
	0		$1.94*10^{7}$					
			$3.85*10^{6}$					
			$1.47*10^{6}$					
			$6.43*10^{4}$					

			$3.86*10^5$					
			$5.57*10^6$					
	$1.14*10^5$				0	0/1		
	$2.96*10^3$							
08/15-16	$1.00*10^4$	5/5						
	1.58							
	$9.65*10^2$							
	$4.75*10^4$				0			
	0				0			
09/19	0	1/5			0	0/5		
	0				0			
	0				0			

GE: genomic equivalent, prevalence = ratio number of Rv positive specimen / total number of specimen tested.
